# Norwegian scabies in a renal transplant patient

**DOI:** 10.4103/0971-4065.65302

**Published:** 2010-04

**Authors:** K. Sampathkumar, A. R. Mahaldar, M. Ramakrishnan, S. Prabahar

**Affiliations:** Department of Nephrology, Meenakshi Mission Hospital and Research Centre, Madurai- 625 107, India; 1 Department of Dermatology, Meenakshi Mission Hospital and Research Centre, Madurai- 625 107, India

**Keywords:** Ivermectin, renal transplant, scabies

## Abstract

A variety of skin infections are encountered in postrenal transplant setting. Though bacterial and fungal infections are more common, surprises are in store for us sometimes. We describe a patient who underwent renal transplant two years ago, presenting with a painless, mildly pruritic expanding skin rash over abdomen. Histological examination of the skin biopsy showed that stratum corneum had multiple burrows containing larvae and eggs of *Sarcoptes scabiei*. The patient was treated with ivermectin 12 mg weekly once for 2 doses along with topical 5% permethrin and permethrin soap bath. There was remarkable improvement in the skin lesions with complete resolution in two weeks. Norwegian or crusted scabies is caused by massive infestation with *Sarcoptes scabiei* var. hominis. It can be rarely encountered in the post-transplant setting, which underscores the importance of early diagnosis and treatment before secondary bacterial infection sets in.

## Introduction

A variety of skin infections can develop in patients after renal transplantation. Bacterial and fungal infections are very common, whereas only isolated case reports describing crusted scabies in renal transplant setting have been published. It is vital to make an early diagnosis of scabies to prevent secondary infection, which can prove fatal due to systemic dissemination after renal transplantation.

## Case Report

**Figure 1:**
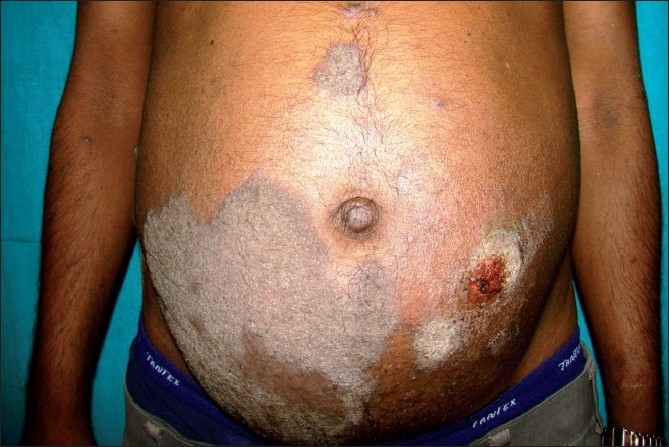
Crusted skin lesions over the abdomen

A 35-year-old patient of autosomal dominant polycystic kidney disease underwent live donor renal transplantation two years ago with a haplomatched donor. He was having stable allograft function. Immunosuppression protocol consisted of cyclosporine, azathioprine and prednisolone. He presented to the outpatient clinic with a large, irregular crusted plaque sized 15 × 9 cm over the abdomen and pubic area. The lesion was painless and minimally pruritic [Figure 1].

**Figure 2:**
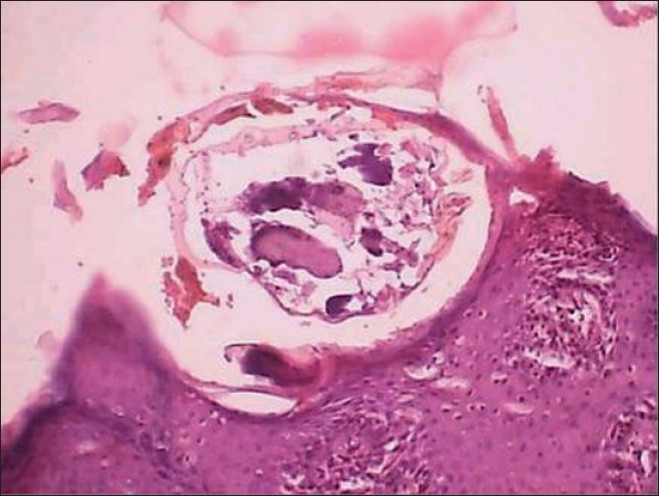
A skin burrow with a larval form of *Sarcoptes scabiei*

Differential diagnosis consisted of fungal infections such as blastomycosis or zygomycosis and tuberculosis. Skin biopsy showed hyperkeratosis, acanthosis and spongiosis of stratum malpighii. Stratum corneum revealed multiple subcorneal burrows containing *Sarcoptes scabiei* larvae and eggs. Dermis had inflammatory cell infiltrates rich in eosinophils and lymphocytes [Figure 2].

The patient was treated with two doses of oral ivermectin 12 mg one week a part along with topical 5% permethrin and permethrin soap bath. There was remarkable improvement in the skin lesions with complete resolution in two weeks. The patient’s family members were counseled, immediate environment sanitized and all household contacts were treated with oral ivermectin and permethrin soap.

## Discussion

Norwegian or crusted scabies is an infrequent, albeit severe, infection caused by massive infestation with *Sarcoptes scabiei* var. hominis. Danielssen and Boeck, who considered the disease to be a form of leprosy endemic to Norway, first described the condition, which at present is known as crusted scabies, in 1848.[1] In 1851, the etiology was correctly attributed to the scabies mite, and Hebra named the disease “Scabies Norvegic Boeckii,” which was later shortened to “Norwegian scabies.” The term Norwegian scabies, even though still in common use, should be discarded, as there is no inherent connection between Norway and Norwegian scabies; the term crusted scabies, as others have suggested, is preferable.[2]

Crusted scabies, a fulminant and highly infectious form, results from a failure of the host immune response to control the proliferation of the scabies mite in the skin, thus causing hyperinfestation. It has also been hypothesized that the crusted response to infection results from a tendency to preferentially mount a Th2 response.[3] A population density of 6,312 mites/g was demonstrated in the dust from bed linen of such a patient.[4] Crusted scabies occurs with increased frequency among patients with leukemia, HIV infection, Down’s syndrome, lepromatous leprosy and diabetes.[4,5]

Transplant recipient patients are at increased risk due to the drug-induced impaired cell-mediated immune response. Failure to recognize and treat crusted scabies in a renal transplant recipient resulted in secondary skin infection and staphylococcal brain abscess, which proved fatal.[6] Early warning regarding the atypical forms of scabies in renal transplant patients were sounded by Wolf *et al*.[7]

Crusted scabies manifests clinically as a psoriasiform and warty dermatosis. It usually presents with crusted and hyperkeratotic plaques or nodules in an acral distribution. Erythematous to gray scaly eruptions, often with thick crusts, may also occur on the face, neck, scalp and trunk. In contrast to conventional scabies, the eruptions are typically far less pruritic and classical erythematous papules and burrows may be limited, absent or obscured by the thick crust.[8] On presentation, the disease may be localized or generalized.

The diagnosis of crusted scabies is based on a constellation of clinical findings and identification of the scabies mites, eggs or feces, which are present in skin taken from under the fingernails (hyponychium), web spaces of the fingers or at the end of burrows and examined in a drop of 10% potassium hydroxide or mineral oil on a microscope slide. Other novel methods include videodermatoscopy, epiluminescence microscopy and use of the polymerase chain reaction. When the diagnosis is unexpected, confirmation has been reached by a biopsy, revealing mites burrowing in the stratum corneum.[8,9]

Treatment aims to eradicate the mites, control symptoms and prevent secondary infection. The topical scabicides (sulfur compounds, benzyl benzoate, crotamiton, lindane, malathion, permethrin) used for classical scabies are also effective in crusted scabies. Repeated applications are usually required, and clearing is slower compared to ordinary scabies.[8] Topical permethrin, a synthetic pyrethroid formulation in a 5% cream, is presently the preferred topical scabicidal agent used in combination with oral ivermectin.

Ivermectin has been used successfully in crusted scabies both as monotherapy or in combination with topical scabicides. The therapy may be effective after a 200 *µ*g/kg single dose, but multiple doses are usually required to achieve cure.[10] The risk of bacterial colonization and septicemia should be anticipated in all patients, and secondary infections should be treated aggressively with broad-spectrum antibiotics. The immediate environment of an infected patient may be heavily infested with a large number of mites, with risk of contagion, particularly bedding and clothing. It is recommended that staff avoid skin-to-skin contact, use gloves and gowns and carefully wash the patient’s clothes and towels. Prophylactic treatment of contact may involve an entire institution or visitors and family members.

## Conclusions

This case report illustrates the importance of precisely identifying crusted scabies in renal transplant patients. Scabicidal therapy with ivermectin is curative.
